# Leisure-Time Physical Activity Is Associated With Reduced Risk of Dementia-Related Mortality in Adults With and Without Psychological Distress: The Cohort of Norway

**DOI:** 10.3389/fnagi.2018.00151

**Published:** 2018-05-25

**Authors:** Ekaterina Zotcheva, Geir Selbæk, Espen Bjertness, Linda Ernstsen, Bjørn H. Strand

**Affiliations:** ^1^Department of Public Health and Nursing, Faculty of Medicine and Health Sciences, Norwegian University of Science and Technology, Trondheim, Norway; ^2^Norwegian National Advisory Unit on Ageing and Health, Vestfold Hospital Trust, Tønsberg, Norway; ^3^Center for Old Age Psychiatric Research, Innlandet Hospital Trust, Ottestad, Norway; ^4^Faculty of Medicine, University of Oslo, Oslo, Norway; ^5^Department of Chronic Diseases and Ageing, Norwegian Institute of Public Health, Oslo, Norway

**Keywords:** physical activity, depression, anxiety, cognitive health, brain health, dementia, psychological distress

## Abstract

**Background:** Leisure-time physical activity (PA) has been proposed as a protective factor against dementia, whereas psychological distress is associated with an increased risk of dementia. We investigated the associations of leisure-time PA and psychological distress with dementia-related mortality, and whether the association between leisure-time PA and dementia-related mortality differs according to level of psychological distress.

**Methods:** 36,945 individuals from the Cohort of Norway aged 50-74 years at baseline (1994–2002) were included and followed up until January 1st 2015. Leisure-time PA and psychological distress were assessed through questionnaires, whereas dementia-related mortality was obtained through the Norwegian Cause of Death Registry. Adjusted Cox regression analyses were used to estimate hazard ratios (HR) and 95% confidence intervals (95%CI).

**Results:** Compared to inactivity, leisure-time PA was associated with a decreased risk of dementia-related mortality; low intensity leisure-time PA (HR = 0.73, 95% CI 0.59–0.89); high intensity leisure-time PA (HR = 0.61, 95%CI 0.49-0.77). A statistically significant difference in dementia-related mortality risk was observed between low and high intensity leisure-time PA (*p* < 0.05). Psychological distress was associated with an increased risk of dementia-related mortality (HR = 1.45, 95% CI 1.16–1.81). Among non-distressed, leisure-time PA was associated with a decreased dementia-related mortality risk; low intensity leisure-time PA (HR = 0.77, 95% CI 0.61–0.97); high intensity leisure-time PA (HR = 0.65, 95% CI 0.51–0.84). The same applied for those with psychological distress; low intensity leisure-time PA (HR = 0.57, 95% CI 0.35–0.94); high intensity leisure-time PA (HR = 0.42, 95% CI 0.22–0.82). The interaction between leisure-time PA and psychological distress on dementia-related mortality was not statistically significant (*p* = 0.38).

**Conclusions:** Participating in leisure-time PA was associated with a reduced risk of dementia-related mortality, whereas psychological distress was associated with an increased risk of dementia-related mortality. Leisure-time PA appears to be equally strongly related with dementia-related mortality among those with and without psychological distress, underlining the importance of leisure-time PA for various groups of middle-aged and older adults.

## Introduction

Physical activity (PA) is widely considered one of the key lifestyle factors associated with reduced risk of mortality (Arem et al., 2015) and a number of non-communicable diseases (Reiner et al., 2013). Furthermore, a large body of research indicates that participating in regular PA may reduce the risk of cognitive decline (Blondell et al., 2014) and dementia (Rosness et al., 2014; Guure et al., 2017). A recently published meta-analysis of prospective studies demonstrated a dose-response relationship between volume and intensity of PA and risk of dementia (Xu et al., 2017). For every 10 metabolic equivalent of task hours (MET-h) increase per week, there was a 10% decrease in the risk of all-cause dementia (Xu et al., 2017). As populations across the globe age, the prevalence of dementia is predicted to increase substantially, from approximately 47 million in 2015, to 132 million by 2050 (Prince et al., 2015). Hence, measures to prevent or delay new cases of dementia are required, and research on protective and risk factors for dementia has intensified during the last decade (Livingston et al., 2017).

Both early- and late-life depression has been linked to an increased risk of dementia (Byers and Yaffe, 2011). Likewise, psychological distress, characterized by general symptoms of anxiety and depression, has been associated with a higher risk of dementia (Skogen et al., 2015) and dementia-related mortality (Rosness et al., 2016). However, PA has repeatedly been associated with reduced symptoms of depression and anxiety in non-clinical populations (Rebar et al., 2015). A recent randomized exercise trial revealed that increases in moderate-to-vigorous PA predicted reductions in psychological distress in older adults (Awick et al., 2017), and a meta-analysis of randomized controlled studies showed that exercise has a significant antidepressant effect among individuals with depression (Schuch et al., 2016). Thus, existing research indicates that PA is beneficial for both mental and cognitive health.

Despite the aforementioned associations between PA, psychological distress, and dementia, little is known on how PA is associated with dementia risk among individuals with psychological distress. Khatri et al. (2001) randomized clinically depressed older adults to either an anti-depressive medication group, an aerobic exercise group, or a combined exercise and medication group. After 4 months, the researchers found that aerobic exercise improved cognitive function among depressed older adults, and that these improvements corresponded to decreases in depressive symptoms (Khatri et al., 2001). However, the latter study did not investigate risk of incident dementia. The aim of the present study was to investigate the associations of leisure-time PA and psychological distress with dementia-related mortality in middle-aged and older adults, and to examine whether the association between leisure-time PA and dementia-related mortality differs according to level of psychological distress.

## Methods

### Study population

The present study is part of the Gene-Environment Interaction in Dementia (GENIDEM) project, which aims to investigate environmental and genetic factors for dementia. The study population comprised 36,945 individuals free from symptoms or diagnosis of heart disease from the Cohort of Norway (CONOR) aged 50–74 years at baseline (1994–2002) linked with the Norwegian Cause of Death Registry and the National Education Database by means of a unique personal identification number. CONOR is a multipurpose study set up to study aetiological factors for a large variety of diseases, and includes 10 epidemiological cohorts from different geographical areas in Norway (Naess et al., 2008). In the present study, three epidemiological cohorts were excluded, as they did not include the CONOR Mental Health Index (CONOR-MHI) scale used to assess psychological distress.

Baseline data collection in CONOR was carried out from 1994 to 2002, following a standardized procedure, where participants received a letter of invitation containing an information brochure by mail 2 weeks prior to a health examination. The health examination included a physical checkup including measurements of participants' height, weight, and blood pressure, as well as drawing blood samples. After completing the examination, the participants returned a self-report questionnaire assessing health- and lifestyle-related variables by mail (Naess et al., 2008). Study participants were followed from baseline until death, emigration, or January 1st 2015, whichever occurred first, with a maximum follow-up time of 20.4 years (mean 15.3). Mean age at follow-up was 76.5 years (max 94.1 years).

All participants included in CONOR gave their written informed consent. The participants' names and personal ID numbers were omitted before data were made available for research purposes. The study was approved by the Norwegian Data Inspectorate and the Regional Committees for Medical Research Ethics, and was conducted in accordance with the Declaration of Helsinki.

### Physical activity

Participants were asked to define the intensity and duration of their leisure-time PA in an average week during the past year. The leisure-time PA question was divided into intensity categories of “low intensity” (not causing perspiration or panting) and “high intensity” (causing perspiration and/or panting) leisure-time PA, each with four alternatives related to average hours per week: “none,” “ < 1h,” “1–2 h,” and “≥3 h.” To ensure sufficient statistical power, all individuals participating in any low intensity leisure-time PA were placed in one group and all individuals participating in any high intensity leisure-time PA were placed in one group, irrespective of hours of weekly leisure-time PA. Participants who replied “none” to both categories of leisure-time PA were considered inactive. Participants who replied “none” on one category of but did not provide an answer to the other category of leisure-time PA, or who had missing answers on both categories were excluded. In the present study, participants were categorized into the following three categories of leisure-time PA based on intensity: “inactive,” “low intensity leisure-time PA,” and “high intensity leisure-time PA.”

### Psychological distress

Psychological distress was assessed with the CONOR Mental Health Index (CONOR-MHI). CONOR-MHI consists of seven items assessing general symptoms of depression and anxiety, and is based on a modification of the General Health Questionnaire (GHQ) and the Hopkins Symptom Check List (HSCL-10) (Søgaard et al., 2003). The items on the CONOR-MHI are shown below. Each item has four answer categories: “no,” “a little,” “a good amount,” and “very much,” and are given values 1–4. The CONOR-MHI score is calculated by dividing the total score (range 7–28) on all seven items by seven, resulting in a range of 1–4, where 1 represents low level of psychological distress, and 4 represents high level of psychological distress. In records containing one missing value, the value was replaced with the sample mean value for each item, whereas records with two or more missing items were excluded.

CONOR Mental Health Index
Have you, in the course of the last two weeks, felt:
Nervous and unsettled?
Troubled by anxiety?
Secure and calm (inverse score)?
Irritable?
Happy and optimistic (inverse score)?
Sad/depressed?
Lonely?

A study comparing the CONOR-MHI to the HSCL-10 and the Hospital Anxiety and Depression Scale (HADS) showed a strong correlation with both scales, *r* = 0.70 and *r* = 0.76, respectively (Søgaard et al., 2003). The same study showed that a cut-off at ≥2.15 on the CONOR-MHI has a sensitivity and specificity of, respectively, 41 and 98% for caseness of HADS-anxiety, 38 and 96% for HADS-depression, and 66 and 95% for HSCL-10 (Søgaard et al., 2003). Based on the cut-off of ≥2.15 on the CONOR-MHI, participants in the present study were categorized into two groups: “no psychological distress” or “psychological distress.”

### Dementia-related mortality

Dementia-related mortality was used as a proxy for dementia illness, and was obtained from death certificates from the Norwegian Cause of Death Registry (Rosness et al., 2014). Dementia was identified when it served either as the underlying, immediate, or accompanying cause of death, according to the International Statistics Classification of Diseases and Related Health Problems, 10th revision, codes F00-F03 and G30.0-G30.9. In the present study population, a total of 919 dementia-related deaths were registered during the follow-up period. For validity purposes, we re-ran analyses with cases restricted to those with dementia as underlying cause.

### Covariates

The following variables that could possibly affect the association between leisure-time PA, psychological distress, and dementia-related mortality were identified based on prior studies: sex, education, diabetes, smoking, body mass index (BMI; weight in kilograms divided by height in meters squared), and hypertension. Demographic and health-related covariates were obtained from the results of the physical checkup and the self-report questionnaire at baseline. Attained educational level was obtained by coupling the participants' unique identification number to the National Education Database. The education variable was then divided into three groups: “high” (university degree/college, corresponding to 13 or more years of schooling), “medium” (secondary qualifications, corresponding to 10 years of schooling), and “low” (elementary school, corresponding to 7 years of schooling) (Strand et al., 2014). Participants who reported currently or previously suffering from diabetes were categorized as diabetic. Smoking habits were dichotomized into daily or non-daily smoker. BMI was divided into four categories: underweight (BMI < 18.5), normal weight (BMI 18.5–24.9), overweight (BMI 25–29.9), and obesity (BMI ≥30). Participants were categorized as hypertensive at a systolic pressure of ≥160 mm Hg and/or a diastolic pressure of ≥100 mm Hg, according to the National Institutes of Health guidelines (National Institutes of Health, 2015).

### Statistical analyses

Adjusted Cox proportional hazards regression was used to estimate hazard ratios (HR) and 95% confidence intervals (95% CI) for the associations of leisure-time PA and psychological distress with dementia-related mortality. Attained age was used as the time variable in the regression models, and thereby all the models were finely adjusted by age. Emigration or non-dementia-related mortality were censored. For leisure-time PA, the “inactive” group served as the reference group, whereas for psychological distress, “no psychological distress” served as the reference group. Leisure-time PA served as the main exposure in the regression models. In the first regression model (model 1), the analyses were adjusted for sex. Next, education, smoking, and psychological distress were added to the model (model 2). The final model (model 3) included the covariates from model 1 and 2, in addition to diabetes, BMI, and hypertension.

To investigate whether psychological distress modified the association between leisure-time PA and dementia-related mortality, an interaction term between leisure-time PA and psychological distress was added to the regression models. In addition, relative excess risk due to interaction (RERI) estimates were calculated to investigate possible additive interaction between leisure-time PA and psychological distress. To obtain psychological distress-specific associations between leisure-time PA and dementia-related mortality, the regression models 1, 2, and 3 were stratified by psychological distress. In these analyses, psychological distress was removed as a covariate from the regression models. Stata version 14 was used for all statistical analyses.

## Results

Baseline characteristics of the study sample by leisure-time PA group are presented in Table 1. Participants in the leisure-time PA groups had higher education, less psychological distress, hypertension, and diabetes, and were less likely to smoke compared to inactive participants. The high intensity leisure-time PA group consisted of less women than the low intensity leisure-time PA group and the inactive group.

**Table 1:** Baseline characteristics of study sample by leisure-time physical activity group.

	**Inactive^a^**	**Low intensity leisure-time PA^b^**	**High intensity leisure-time PA^c^**
Age at survey, mean (SD), y	62.7 (7.1)	61.8 (6.9)	60.2 (6.6)
Age at exit, mean (SD), y	76.7 (7.0)	77.2 (6.9)	75.7 (6.5)
Women, no. (%)	1,594 (54.1)	10,758 (58.4)	6,067 (38.9)
Higher education >1 y, no. (%)	136 (4.6)	2,221 (12.1)	3,672 (23.6)
Psychological distress^d^, no. (%)	395 (13.4)	1,452 (7.9)	889 (5.7)
CONOR-MHI score, mean (SD)	1.62 (0.52)	1.52 (0.43)	1.48 (0.40)
Hypertension, no. (%)	790 (26.8)	4,350 (23.6)	2,950 (18.9)
Daily smoking, no. (%)	1,180 (40.1)	5,454 (29.6)	3,767 (24.2)
Diabetes, no. (%)	154 (5.2)	775 (4.2)	507 (3.3)
BMI, mean (SD)	27.9 (5.1)	26.9 (4.2)	26.6 (3.7)
All-cause mortality, no. (%)	1,281 (43.5)	5,675 (30.8)	3,372 (21.6)
Dementia-related mortality, no. (%)	114 (3.9)	536 (2.9)	269 (1.7)

PA, physical activity; CONOR-MHI, Cohort of Norway Mental Health Index; BMI, body mass index (calculated as weight in kilograms divided by height in meters squared).

a n = 2,944.

b n = 18,412.

c n = 15,589.

d Psychological distress CONOR-MHI ≥ 2.15.

Leisure-time PA was associated with a decreased risk of dementia-related mortality when compared to inactivity; low intensity leisure-time PA (HR = 0.68, 95% CI 0.56–0.84); high intensity leisure-time PA (HR = 0.54, 95% CI 0.43–0.68) in a model adjusted by sex (Table 2, Model 1). The results were slightly attenuated, but remained statistically significant after additional adjustment for education, smoking and psychological distress; low intensity leisure-time PA (HR = 0.76, 95% CI 0.62–0.93); high intensity leisure-time PA (HR = 0.63, 95% CI 0.50–0.80) (Model 2). Additional adjustment for diabetes, hypertension and BMI did not attenuate the results noteworthy; low intensity leisure-time PA (HR = 0.73, 95% CI 0.59–0.89); high intensity leisure-time PA (HR = 0.61, 95% CI 0.49–0.77) (Model 3). A statistically significant difference in dementia-related mortality hazard ratios was observed between low and high intensity leisure-time PA (*P* < 0.05). Psychological distress was associated with an increased risk of dementia-related mortality (HR = 1.45, 95% CI 1.16–1.81) in the fully adjusted model 3.

**Table 2:** Dementia-related mortality by level of leisure-time physical activity.

	***n***	**DRM cases**	**HR (95% CI) Model 1^a^**	**HR (95% CI) Model 2^b^**	**HR (95% CI) Model 3^c^**
Total	36,945	919			
Inactive	2,944	114	1.00	1.00	1.00
Low intensity leisure-time PA	18,412	536	0.68 (0.56–0.84)	0.76 (0.62–0.93)	0.73 (0.59–0.89)
High intensity leisure-time PA	15,589	269	0.54 (0.43–0.68)	0.63 (0.50–0.80)	0.61 (0.49–0.77)

DRM, dementia-related mortality; HR, hazard ratio; 95% CI, 95% confidence interval; PA, physical activity.

a Adjusted for sex.

b Adjusted for sex, education, smoking, and psychological distress.

c Adjusted for sex, education, smoking, psychological distress, diabetes, BMI, and hypertension.

### Stratified analyses of leisure-time PA and dementia-related mortality by psychological distress

To investigate whether the association between leisure-time PA and dementia-related mortality varied according to psychological distress status, we performed analyses stratified by psychological distress. Among non-distressed, leisure-time PA was associated with a decreased risk of dementia-related mortality when compared to inactivity in a model adjusted by sex; low intensity leisure-time PA (HR = 0.75, 95% CI 0.60–0.95); high intensity leisure-time PA (HR = 0.60, 95% CI 0.47–0.77) (Table 3, upper panel, Model 1). The results were slightly attenuated after further adjustment, but remained statistically significant (Table 3, upper panel, Models 2 and 3).

**Table 3:** Dementia-related mortality by level of leisure-time physical activity, stratified by psychological distress.

	***n***	**DRM cases**	**Model 1^a^ HR (95% CI)**	**Model 2^b^ HR (95% CI)**	**Model 3^c^ HR (95% CI)**
Total	36,945	919			
No psychological distress	33,379	830			
Inactive	2,462	87	1.00	1.00	1.00
Low intensity leisure-time PA	16,470	490	0.75 (0.60–0.95)	0.80 (0.64–1.01)	0.77 (0.61–0.97)
High intensity leisure-time PA	14,447	253	0.60 (0.47–0.77)	0.67 (0.52–0.86)	0.65 (0.51–0.84)
Psychological distress	2,647	89			
Inactive	368	27	1.00	1.00	1.00
Low intensity leisure-time PA	1,406	46	0.54 (0.33–0.87)	0.60 (0.37–0.98)	0.57 (0.35–0.94)
High intensity leisure-time PA	873	16	0.42 (0.22–0.80)	0.46 (0.24–0.89)	0.42 (0.22–0.82)

DRM, dementia-related mortality; HR, hazard ratio; 95% CI, 95% confidence interval; PA, physical activity.

a Adjusted for sex.

b Adjusted for sex, education, and smoking.

c Adjusted for sex, education, smoking, diabetes, BMI, and hypertension.

Similarly, among distressed participants, in a sex adjusted model (Table 3, lower panel, Model 1), leisure-time PA was associated with a decreased risk of dementia-related mortality when compared to being inactive; low intensity leisure-time PA (HR = 0.54, 95% CI 0.33–0.87); high intensity leisure-time PA (HR = 0.42, 95% CI 0.22–0.80). Further adjustment did not attenuate these HRs much, and the associations remained statistically significant (Table 3, lower panel, Models 2 and 3). Although the decrease in dementia-related mortality risk appeared to be larger for the psychological distress group than for participants without psychological distress, there was no statistically significant multiplicative interaction between leisure-time PA and psychological distress on dementia-related mortality (*P* = 0.38). Furthermore, there was no statistically significant additive interaction between leisure-time PA and psychological distress on dementia-related mortality, RERI estimate = 0.155 (95% CI −0.80 to 1.11).

## Discussion

The results of the present study of 36,945 middle-aged and older adults demonstrated that participating in low or high intensity leisure-time PA was associated with a lower risk of dementia-related mortality, compared to being inactive. Further, psychological distress was associated with an increased risk of dementia-related mortality, whereas analyses stratified by psychological distress revealed that participating in both low and high intensity leisure-time PA was associated with a lower risk of dementia-related mortality among both those with and without psychological distress. However, we did not observe a statistically significant interaction between leisure-time PA and psychological distress on dementia-related mortality.

Prior studies have found an association between higher levels of PA and lower risk of dementia (Blondell et al., 2014; Guure et al., 2017; Xu et al., 2017). The present study builds further on these findings, by demonstrating the importance of the intensity of leisure-time PA for reducing the risk of dementia-related mortality. Our results showed that participating in high intensity leisure-time PA was associated with a significantly lower risk of dementia-related mortality than low intensity leisure-time PA, suggesting that the intensity of PA may be an essential factor in reducing the risk of dementia-related mortality. This is in line with an earlier longitudinal twin study, in which Iso-Markku and colleagues found that persistent participation in vigorous PA was associated with reduced risk of dementia mortality (Iso-Markku et al., 2015). In the study by Iso-Markku and colleagues (Iso-Markku et al., 2015), vigorous PA was defined based on type of activity, whereas our study includes a relative measure of intensity, with high intensity leisure-time PA defined as taking part in PA that leads to panting and perspiration. A study found that 5.4 million individuals aged 24–65 residing in England would exceed 70% maximum heart rate by walking at approximately 4.8 km/h (Kelly et al., 2001). In absolute intensity terms, walking at this pace is typically classified as a light intensity activity (Ainsworth et al., 2000), even though an individual's body may be working at a higher intensity. Thus, considering perceived effort of leisure-time PA provides a different measure than considering absolute intensity based on type of activity. It is important to note that our study demonstrated a significant reduction in risk of dementia-related mortality for both low and high intensity leisure-time PA, compared to being inactive. Hence, the public health emphasis should be on finding ways to motivate inactive individuals to participate in any kind of leisure-time PA.

Our results further demonstrated that psychological distress was associated with an increased risk of dementia-related mortality. This finding is in accordance with an earlier CONOR study showing higher risk of dementia-related mortality among psychologically distressed individuals (Skogen et al., 2015), as well as studies on other populations indicating that depression increases risk of dementia (Byers and Yaffe, 2011). Whether participating in leisure-time PA is associated with the risk of dementia among individuals with psychological distress has not been widely examined. The results of the present study indicate that both low and high intensity leisure-time PA can significantly reduce the risk of dementia-related mortality among individuals with psychological distress. However, our analyses of both multiplicative and additive interaction did not indicate any interaction between psychological distress and leisure-time PA on risk of dementia-related mortality. Nevertheless, the results underline the importance of leisure-time PA for decreasing risk of dementia-related mortality among both psychologically distressed and non-distressed individuals.

A possible explanation for the associations of leisure-time PA and psychological distress with dementia-related mortality may lie within brain imaging studies. Magnetic resonance imaging studies have repeatedly demonstrated that exercise may induce neurogenesis in brain regions important for cognitive health (Colcombe et al., 2006; Erickson et al., 2011), and PA has been associated with preserved hippocampal volume in individuals at high risk for Alzheimer's disease (Smith et al., 2014). Depression, on the other hand, has been linked to brain atrophy (Geerlings et al., 2013; Elbejjani et al., 2015) and poorer cognitive functioning (Bunce et al., 2012; Lebedeva et al., 2015). A longitudinal study with over a decade of follow-up revealed that individuals who were least active had an increased risk of dementia, and that physical activity was positively associated with total brain and hippocampal volume (Tan et al., 2017). Hence, atrophy and neurogenesis in cognitively important brain areas due to PA and psychological distress may be part of the underlying mechanisms behind our results.

The present study has some limitations. Dementia obtained from death certificates has relatively low sensitivity, which may have introduced misclassification (Bjertness et al., 1998). However, dementia on death certificates has a high specificity and positive predictive value (Bjertness et al., 1998). Our endpoint included deaths with dementia as accompanying cause of death. Thus, it is possible that the observed associations between leisure-time PA/psychological distress and the outcome may be due to underlying causes of death other than dementia. However, the results were similar when analyses were carried out only among participants where dementia was the underlying cause of death (*n* = 433, data not shown). In the case of random underreporting of dementia on death certificates, associations would be biased toward the null hypothesis.

Self-reported leisure-time PA may be prone to over- or underrepresentation of intensity as it is based on subjective experience. The same applies to the self-report of psychological distress during the last 2 weeks, where individual scores may have been affected by recent events or daily mood. However, assessing psychological distress in a large sample from the general population rather than using participants with a diagnosis of depression or anxiety makes the results more relevant for public health rather than just clinical populations, which have been the emphasis of many prior studies. Our study does not include longitudinal measures of leisure-time physical activity and psychological distress, which would have provided more information on how these lifestyle factors during the life course are associated with dementia-related mortality. Further, some variables, such as social network, were not available in this study, which may have introduced bias due to unmeasured confounders. Finally, despite the long follow-up time in our study (mean 15.3 years, max 20.4 years), reverse causation cannot be completely ruled out due to the long preclinical period of dementia (Jack et al., 2013).

In conclusion, our study of 36,945 middle-aged and older adults revealed an association between both low and high intensity leisure-time PA and reduced risk of dementia-related mortality. Psychological distress was associated with an increased risk of dementia-related mortality, whereas participants with psychological distress who participated in leisure-time PA had a reduced risk of dementia-related mortality compared to distressed and inactive participants. Whereas prior studies have emphasized either PA or psychological distress as a predictor of dementia, our study incorporates these exposures. In sum, our findings underline the importance of PA for cognitive health in middle-aged and older individuals with and without psychological distress. Health care professionals should encourage physical activity in middle-aged and older adults, particularly among individuals with psychological distress, as they appear to be at a higher risk of incident dementia.

## Data availability

The data analyzed in this study was obtained from the Norwegian Institute of Public Health. Requests to access these datasets should be directed to the Norwegian Institute of Public Health, Oslo, Norway.

## Author contributions

EZ, GS, EB, LE, and BS all contributed to the conceptualization and design of the study, and drafting and revising of the manuscript prior to submission. Statistical analyses were carried out by BS.

### Conflict of interest statement

The authors declare that the research was conducted in the absence of any commercial or financial relationships that could be construed as a potential conflict of interest.

## Abbreviations

PA: physical activity
CONOR: Cohort of Norway
CONOR-MHI: Cohort of Norway Mental Health Index
HSCL-10: Hopkins Symptom Check List
HADS: Hospital Anxiety and Depression Scale
DRM: dementia-related mortality.
